# Promoting empowerment for people living with dementia in nursing homes: Development and feasibility evaluation of an empowerment program

**DOI:** 10.1177/14713012221124985

**Published:** 2022-09-05

**Authors:** Charlotte van Corven, Annemiek Bielderman, Mandy Wijnen, Ruslan Leontjevas, Peter LBJ Lucassen, Maud JL Graff, Debby L Gerritsen

**Affiliations:** Department of Primary and Community Care, 6034 Radboud University Medical Center, Radboud Institute for Health Sciences, Radboud Alzheimer Center, Nijmegen, The Netherlands; Department of Primary and Community Care, 6034 Radboud University Medical Center, Nijmegen, The Netherlands; Faculty of Psychology, 10198 Open University of The Netherlands, Heerlen, The Netherlands; Department of Primary and Community Care, 6034 Radboud University Medical Center, Radboud Institute for Health Sciences, Radboud Alzheimer Center, Nijmegen, The Netherlands; Department of Primary and Community Care, 6034 Radboud University Medical Center, Radboud Institute for Health Sciences, Nijmegen, The Netherlands; Scientific Institute for Quality of Healthcare and Department of Rehabilitation, 6034 Radboud University Medical Center, Radboud Institute for Health Sciences, Radboud Alzheimer Center, Nijmegen, The Netherlands; Department of Primary and Community Care, 6034 Radboud University Medical Center, Radboud Institute for Health Sciences, Nijmegen, The Netherlands

**Keywords:** Dementia, psychosocial support, healthcare professionals, family caregivers, nursing homes

## Abstract

**Objectives:**

This article describes the development and feasibility evaluation of an empowerment program for people living with dementia in nursing homes.

**Methods:**

Development and feasibility evaluation of the empowerment program was guided by the British Medical Research Council’s (MRC) framework. In the developmental phase, we used intervention mapping to develop the theory- and evidence-based intervention. During the feasibility phase, two care teams utilised the program from September to December 2020. We evaluated the feasibility in terms of demand, acceptability, implementation, practicality, integration and limited efficacy.

**Findings:**

This study showed that, according to healthcare professionals, the program was feasible for promoting empowerment for people living with dementia in a nursing home. Healthcare professionals mentioned an increased awareness regarding the four themes of empowerment (sense of identity, usefulness, control and self-worth), and greater focus on the small things that matter to residents. Healthcare professionals experienced challenges in involving family caregivers.

**Conclusion:**

An important step is to take into account the implementation prerequisites that follow from our findings, and to further investigate feasibility, as the use of the program and data collection was hindered by the COVID-19 pandemic. Subsequent research could investigate the effects of the empowerment program.

## Introduction

Healthcare organisations continuously try to improve the quality of care for nursing home residents living with dementia. In recent decades, there has been a shift from task-oriented care, with a focus on illness, to person-centred (Edvardsson et al., 2008; Kitwood, 1997; McCormack et al., 2012; Simmons & Rahman, 2014) and relationship-centred care (Nolan et al., 2004). These are approaches that focus on the whole person and their relationship with caregivers. The concept of empowerment fits with this focus, as it promotes a sense of identity, usefulness, control and self-worth (van Corven et al., 2021a). These four domains of empowerment were identified using focus group discussions and interviews with people living with dementia, their family caregivers and healthcare professionals (van Corven et al., 2021a). An extensive systematic literature review showed that empowerment is a dynamic process, taking place between the person living with dementia and their environment (van Corven et al., 2021b). An empowering approach encourages the person living with dementia to be a person with individual talents and capabilities and may contribute to reciprocity in relationships (Vernooij-Dassen et al., 2011; Westerhof et al., 2014).

Therefore, in nursing homes, the concept of empowerment has received growing attention (Carpenter et al., 2002; Hung & Chaudhury, 2011; Martin & Younger, 2000; Swall et al., 2017; Watt et al., 2019). Nevertheless, interventions that specifically aim at empowering people living with dementia in a nursing home are lacking (van Corven et al., 2022). Such interventions could be valuable as they may help to focus on what is possible, instead of what is no longer possible, by striving to achieve the four themes of empowerment (a sense of identity, usefulness, control and self-worth) in the interaction between people living with dementia and their environment (van Corven et al., 2021b). An important step in improving quality of care for people living with dementia is to develop and test interventions that specifically aim to promote empowerment for those people, and to support (in)formal caregivers in this process.

In this study, we develop such a program (the WINC empowerment program) for people living with dementia in a nursing home. The aim of the program is to reflect and act on the wishes and needs of people with dementia and their family caregivers regarding the four themes of empowerment (van Corven et al., 2021b). It aims to provide concrete opportunities for healthcare professionals and family caregivers to address and support the strengths of the person with dementia, and through this, encourage the person with dementia to increase their sense of self-worth (W), identity (I), usefulness and being needed (N), and control (C). This article describes the development and feasibility evaluation of this WINC empowerment program.

## Materials and methods

### Design

The development and feasibility evaluation of the WINC empowerment program was guided by the British Medical Research Council’s (MRC) framework on how to develop and evaluate complex interventions (Skivington et al., 2021), including four phases: (1) development or identification of the intervention, (2) feasibility, (3) evaluation, and (4) implementation. This article describes phases 1 and 2.

#### Phase 1 of MRC framework: Intervention development

We used intervention mapping (IM) to develop the theory- and evidence-based intervention (Bartholomew et al., 2016). Intervention mapping consists of six different steps: (1) identification of potential improvements (needs assessment), (2) defining the behaviours and their determinants that are needed to reach the improvement goal, (3) selecting behaviour change techniques and ways to apply them, (4) designing the program, (5) specifying an implementation plan, and (6) generating an evaluation plan.

#### Phase 2 of MRC framework: Feasibility

In this phase, we used the method described by Bowen et al. to evaluate the feasibility in terms of demand, acceptability, implementation, practicality, integration, and limited efficacy (Bowen et al., 2009). *Demand* is the extent to which the program is likely to be used; *acceptability* refers to suitability; *implementation* addresses the degree of delivery; *practicality* refers to the extent to which the program is carried out as intended; *integration* relates to the extent to which it can be integrated in existing systems, and *limited efficacy* addresses the promise the program shows of being effective.

### Setting and participants

The program was developed by the project team, consisting of all authors, a quality assurance officer, an elderly care physician from the participating nursing home, and a nursing assistant. The European Working Group of People with Dementia (EWGPWD) and the Alzheimer Associations Academy (AAA) of Alzheimer Europe were consulted on the concept version. In this way, we aimed to ensure that the program reflects the priorities and views of all stakeholders, and that results would be applicable across Europe.

For the feasibility evaluation, we formed a local multidisciplinary working group within the participating nursing home before the start of the program, consisting of the quality assurance officer and elderly care physician (also participating in the project team), a psychologist, two nursing assistants, a specialist nurse, an activity therapist and a researcher (CvC). The contact person of the local multidisciplinary working group (quality assurance officer) approached six care teams from psychogeriatric nursing home units for participation in the study. Three teams were willing to participate. We chose two care teams from the same location for practical reasons, such as reducing travel time for participants and the project team. The three other teams who were not willing to participate did express their interest, but could not participate due to low staffing or high workload.

### Data collection

#### Phase 1 of the MRC framework: Intervention development

Between May 2018 and November 2020, we performed a needs assessment, using focus group discussions and interviews with stakeholders (van Corven et al., 2021a), an integrative literature review (van Corven et al., 2021b), and a European survey to identify existing empowerment interventions (van Corven et al., 2022). Between April and December 2019, regular meetings were held with the project team to discuss and interpret the results of the needs assessment, determine program objectives, and select behaviour change techniques that could be applied in nursing homes. These behaviour change techniques were extracted from the literature (Michie et al., 2011).

In December 2019, three researchers (CvC, AB, DG) joined the annual meetings of the EWGPWD and AAA to present the concept version of the program. Thereafter, we discussed its relevance, potential barriers and possible improvements for 30 min in subgroups.

#### Phase 2 of the MRC framework: feasibility

The study to evaluate the feasibility started in March 2020. The program stopped the same month due to the COVID-19 outbreak, but was restarted and ran between September and December 2020. We collected qualitative and quantitative data regarding feasibility.

##### Qualitative data collection regarding feasibility

We collected qualitative data using the field notes of all meetings with the local multidisciplinary working group, a focus group interview with healthcare professionals from both nursing home units and three members of the multidisciplinary working group (quality assurance officer and two nursing assistants), and telephone interviews with all other members of the multidisciplinary working group and two of the healthcare professionals from both nursing home units. Questions considered the program’s acceptability (e.g., how did you appreciate the program?), implementation and practicality (e.g., what were the barriers and facilitators for using the program?), and limited efficacy (e.g., what did the program bring you and residents?). The focus group interview and individual interviews were moderated by the first author (CvC), tape-recorded and transcribed verbatim. They lasted 1 hour and between 10 and 30 min, respectively. Furthermore, the formulated objectives for each resident were collected from the residents’ personal files.

##### Quantitative data collection regarding feasibility

An overview of the data collection by means of questionnaires is displayed in Table 1. Questionnaires included self-developed statements regarding demand, acceptability, implementation, practicality and integration (see Supplementary Additional File 1). Participants responded to statements on a five-point Likert scale from ‘totally disagree’ to ‘totally agree’. To assess the limited efficacy, we administered standardised questionnaires (see Supplementary Additional File 2).

**Table 1.** Overview of quantitative data collection for healthcare professionals and family caregivers.

#	Moment in time	Feasibility area of focus
Healthcare professionals		
T0	Before start (September 2020)	Overall demand, acceptability and limited efficacy
T1	After end (December 2020)	Overall acceptability, practicality, implementation and limited efficacy
Healthcare professionals proxy about resident living with dementia		
T0	Before start (September 2020)	Limited efficacy
T1	After end (December 2020)	Limited efficacy
Family caregivers		
T0	Before start (September 2020)	Overall demand, acceptability and limited efficacy
T1	After end (December 2020)	Overall acceptability, practicality, implementation and limited efficacy

### Data analysis

For all qualitative data, content analysis was used. Two authors (CvC, MW) coded the text, and constructed categories and themes based on consensus. For the quantitative data, we used medians and ranges to describe the baseline characteristics of participants and outcome measures. We used fractions to describe how many participants agreed with or totally agreed with the statements.

### Ethical considerations

The study was conducted in accordance with Dutch Law and the Declaration of Helsinki. The study protocol was reviewed by the local Medical Ethics Review Committee [CMO Regio Arnhem Nijmegen] (number 2018-4101), which stated that the study was not subject to the Medical Research Involving Human Subjects Act as the participants were not subjected to actions or behavioural rules that were imposed on them. We asked for verbal consent when consulting the EWGPWD and AAA. During the feasibility evaluation, we obtained prior written informed consent from all family caregivers and healthcare professionals. Family caregivers also provided written informed consent for residents living with dementia. The study was registered in the Dutch Trial Register, number (number NL8829).

### Findings

#### Phase 1: Intervention development

Following the first step of intervention mapping, which is identifying potential improvements, we found that for people living with dementia to feel empowered, a sense of identity, usefulness, control and self-worth are important (van Corven et al., 2021a). These four domains of empowerment followed from focus group discussions and individual interviews with people living with dementia (n = 15), family caregivers (n = 16) and healthcare professionals (n = 46), exploring perspectives on empowerment, and needs and wishes regarding an empowerment intervention. Moreover, an extensive systematic literature review showed that empowerment is a dynamic process, with it taking place within the interaction of the person living with dementia and their environment (van Corven et al., 2021b). The European survey showed that stakeholders considered a broad range of interventions empowering in dementia care and research (van Corven et al., 2022). However, none of the available interventions in this survey were specifically developed for or aimed at empowerment. Therefore, the project team concluded that in order to promote empowerment, it is essential to develop and test interventions with a specific focus on empowerment.

Thereafter, for step 2, the project team identified the determinants of behaviours needed to promote empowerment, including knowledge (knowing how to promote empowerment), attitude (recognising the advantages of promoting empowerment), outcome expectations (expecting that promoting empowerment will increase well-being), skills (demonstrating the ability to promote empowerment) and self-efficacy (expressing confidence in the ability to promote empowerment), which is visualised in the model of change (see Supplementary Additional File 3). The behaviour change matrix shows the specific behaviours that people living with dementia and their environment can perform to promote empowerment (see Supplementary Additional File 3).

The selected behaviour change techniques, specified in step 3, included action planning, barrier identification, and focusing on past successes, among others. The techniques may promote the behaviours that people living with dementia and those in their environment can perform to promote empowerment. For example, we thought that healthcare professionals discussing the barriers to promoting empowerment, and ways of overcoming them during the Empowerment Café would be beneficial as it may increase the self-efficacy of the healthcare professionals to promote empowerment for residents, and their outcome expectations. An extensive overview of the behaviour change techniques, literature about these techniques, and how these are applied in the program can be found in Supplementary Additional File 4.

**Figure 1.**
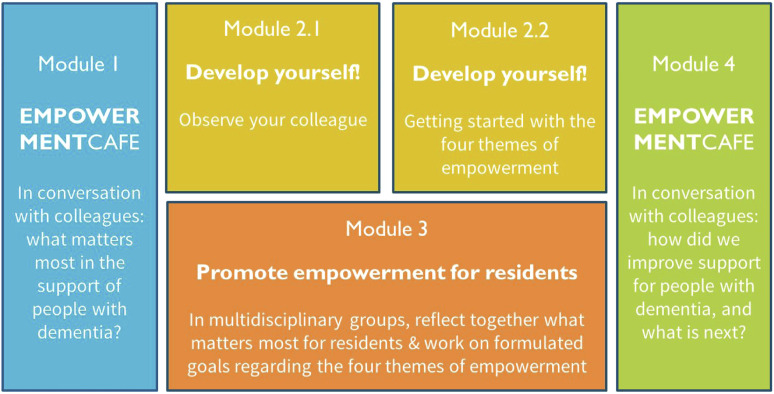
Visual representation of the modules of WINC

For step 4, concerning design of the program, the project team specified the aim of the empowerment program, which is to enable professional caregivers to reflect and act on the wishes and needs of people with dementia and their family caregivers regarding the four themes of empowerment. An overview of the program is displayed in Figure 1.

The project team ensured that the design was suitable for all stakeholders. Module 1 is a kick-off meeting (called the Empowerment Café) in which participating care teams discuss the themes of empowerment, experiences, benefits, barriers, and strategies to overcome these barriers to promotion of empowerment. Here, the project team explains the rest of the WINC empowerment program. For module 2, healthcare professionals work on two exercises for their own professional development. In the first exercise, each member of the care team joins a colleague for 4 hours to observe how they work with respect to empowerment. In the second exercise, each healthcare professional focuses on the themes of empowerment for all residents during their shifts. In the third module, which runs concurrently with the second module, healthcare professionals talk in a small multidisciplinary group about specific residents with the help of the WINC reflection cards, which contain questions about each theme of empowerment. These reflections result in goal setting for all residents, that will be discussed (and adjusted) with the family and the resident (when possible), and evaluated after 6 weeks. Module 4 is a final meeting (again called the Empowerment Café) to share experiences and evaluation of the program and its results, both for healthcare professionals and for residents, and make agreements as to how to continue, for example by repeating some of the modules of the program in the future.

For step 5, regarding specification of an implementation plan, we formed a local multidisciplinary working group within the healthcare organisation to adjust the empowerment program to fit the local setting, for example by discussing how meetings for module 3 could best be organised so that they fit the agenda of most of the healthcare professionals. For step 6, we generated an evaluation plan. This included a description of the used methods to evaluate feasibility, and their time planning.

#### Phase 2: Feasibility

We present data collected between September and December 2020. An overview of the collected data at the initial start in March 2020 can be found in Supplementary Additional Files 4 and 5. Results on the specific modules can be found in Supplementary Additional File 5.

### Participant characteristics

In total, 14 residents, 13 family caregivers and 18 healthcare professionals of two psychogeriatric nursing home units participated in the feasibility study. Quantitative data were collected from all residents, family caregivers and healthcare professionals, while for the qualitative data, seven healthcare professionals participated in the focus group interview and five healthcare professionals in the individual interviews.

The 14 participating residents had a median age of 85 years (range: 67–95 years) and the majority were female (n = 10). The median amount of time residents lived in the nursing home was 2.4 years (range: 0.2–5.3 years). The majority of residents were diagnosed with Alzheimer’s disease (n = 12), others had vascular dementia (n = 1), or a combination of those (n = 1). Residents were married (n = 6), divorced (n = 2) or widowed (n = 5). Data was missing on age (n = 1) and years spent living in the nursing home (n = 2), respectively. The 13 participating family caregivers had a median age of 67 years (range: 51–87 years) and approximately half were female (n = 6). They were either partners (n = 6), siblings (n = 2) or children (n = 5) of the residents, and spent time with the resident once every 2 weeks (n = 2), once a week (n = 1), multiple times a week (n = 8) or every day (n = 2).

The 18 participating healthcare professionals had a median age of 55 years (range: 29–63 years) and the majority were female (n = 17). Their median work experience in healthcare was 25 years (range: 2–46 years), with 15 years specifically on people living with dementia (range: 0.5–41 years), and 13 years within this organisation (range: 1–33 years). They worked as nursing assistants (n = 13), or as a nurse, specialist nurse, well-being coach, psychologist and quality assurance officer (n = 1 for all occupations). Age and years of work experience was missing for two participating healthcare professionals. In both participating psychogeriatric nursing home units, eight people reside. At both units have been working the same psychologist, specialist nurse, well-being coach and quality assurance officer. Six and eight nurses and nursing assistants have been working in the two teams respectively. No information was collected about the working hours of these nurses and nursing assistants.

#### Demand

##### Expectations before the start

In questionnaires prior to the start, almost all healthcare professionals who filled in the questionnaire indicated that they would like to work with WINC, and had the impression this was the same for their colleagues (7/8).

#### Acceptability

##### Expectations before the start

Similarly, in questionnaires prior to the start, all eight healthcare professionals who filled in the questionnaire indicated that their first impression of WINC was good, and they were motivated by WINC. Almost all of the healthcare professionals expected to enjoy working with WINC together with their colleagues, and expected WINC to be of value to their work, and for the care and support of the residents living with dementia (7/8).

Half of the family caregivers who filled in the questionnaire indicated that their first impression of WINC was good (5/10), while the majority thought that it was a good idea that the nursing home would work with WINC (8/10), and that WINC would be of value for the care and support of their family member (7/10).

##### Experiences using WINC

In the questionnaires at the end of the WINC empowerment program, half of all healthcare professionals indicated that they enjoyed working with WINC, that it was of value to their work, and that they would advise other teams of the care organisation to work with WINC (4/8). Further, just under half thought it was of value for the care and support of residents living with dementia, or indicated that they would like to keep using WINC in the future (3/8). The experiences of healthcare professionals varied, as illustrated by these two quotes from healthcare professionals:

This is how you want to look at the resident: replenish what needs and wishes are. I see the added value and I hope we can make the WINC feeling our own. (20)

The project wasn’t of added value for me. I think we as colleagues reflect a lot, and pay attention to our attitude towards residents. I don’t think WINC will add to that. (07)

In the follow-up questionnaires at the end, almost half of the family caregivers indicated that they were well informed about WINC (3/7), that they felt involved enough (4/7), while only a minority indicated that WINC had been of value for the care and support of their loved one (2/7).

### Implementation

#### Experiences

From the qualitative analyses, the following themes emerged regarding implementation: barriers to promoting empowerment, and challenges involving family caregivers.

##### Theme: ‘Barriers to promoting empowerment’

One of the key themes that emerged was ‘barriers to promoting empowerment’. This included (1) COVID-19 measures, and (2) a lack of time. Healthcare professionals mentioned in the interviews that these were barriers to implementing WINC in their daily work, as it hindered undertaking activities or giving attention to individual residents.

I think it is a nice program, but really putting it into practice… I don’t think we now have the time to really put it into practice. (20)

For example, to promote empowerment, healthcare professionals stated the importance of one-to-one activities. They indicated this was sometimes not possible due to other tasks, or they felt they were not giving enough attention to other residents.

That can be difficult. We have four ladies sitting at a table, with whom you can do an activity together. But when doing that, you have in the back of your mind that you are failing the others. I sometimes find that very difficult. (9)

Healthcare professionals reported that this lack of time also caused them to take over tasks residents were performing, while to promote empowerment, they said it would be best if the residents completed the task themselves and therefore attained a sense of usefulness:

Peeling potatoes or something, I sometimes do it myself quickly. Otherwise [with residents] you could be busy with it for almost 45 min. So if it’s busy, I just do it myself. (14)

One healthcare professional mentioned during the interview that she thinks it is very important for the team to direct each other’s attention to the themes of empowerment, as otherwise new activities and attitudes regarding empowerment are easily forgotten.

##### Theme: ‘Challenges involving family caregivers’

Another key theme that emerged was challenges in involving the family caregivers in WINC. Healthcare professionals reported having spoken to family caregivers about the specific goals which were formulated during the multidisciplinary meeting, but family caregivers often responded that the goals were suitable, and did not have any additional feedback.

In theory it seems very nice [involvement of family], but in practice it is just very difficult. (14)

Nevertheless, healthcare professionals reported seeing value in involving family caregivers in WINC, although this differed between family caregivers.

But yeah, if people don’t want to, it just stops. But I do think that if the family takes the time, they will have more ideas. (21)

It also depends on the type of family. If there are four sons who all live far away or find the behaviour of their mother difficult, that will be very different. It depends on how people are in it. (05)

Healthcare professionals reported COVID-19 measures to be a barrier for family involvement, as visits mostly took place in resident’s apartments, and there were fewer informal meetings between family caregivers and healthcare professionals. To improve the involvement of family caregivers, healthcare professionals suggested changing their attitudes to family caregivers from the nursing home placement onwards:

But yeah, we also need to have a different attitude. When family visits say ‘your father is coming to live here, but we also expect something of you’. I think we need to move in that direction in the future. (05)

### Practicality

#### Expectations before the start

From the questionnaires prior to the start of the program, less than half of all healthcare professionals indicated feeling that they would have enough time to work with WINC (3/8), and all eight indicated that it was clear to them what would be expected.

#### Experiences

During the follow-up questionnaires, less than half indicated that they felt they had enough time to work with WINC (3/9). Furthermore, the majority indicated it was clear what was expected of them (7/9).

In the interviews, participants reported that they perceived the four themes of empowerment to be overlapping, for example when making goals for residents or answering the questions in their personal booklet.

What I noticed when forming the specific goals for residents, it was sometimes difficult to tell what was exactly meant by a theme. […] a lot of things overlapped. (03)

### Integration

#### Experiences

During the interviews, healthcare professionals mentioned that WINC suited their way of working. Many interviewees mentioned that the themes of empowerment were not new to them, but WINC helped direct attention to this way of working.

I saw it more as an addition, to refresh again. It provided a moment to stop and think about what we are doing. (25)

They mentioned disliking that meetings for WINC were in addition to their normal working hours, which meant they had to come to work in their free time. Nevertheless, this is also the case for other projects.

#### Limited efficacy

From the qualitative analyses, the following theme emerged regarding limited efficacy: added value of WINC.

##### Theme: ‘Added value of WINC’

One of the key themes that emerged from the focus group discussions and interviews was the added value of WINC. During the interviews, healthcare professionals indicated that the greatest benefit of WINC was raising awareness regarding the four themes of empowerment. They reported that reflecting on their way of working broke through their regular and established routines:

We already do a lot, so the program only helped to make me more aware. For example, one of our residents can make a lot of choices himself, you become aware of that again. I have to be honest that the program didn’t have any added value, except this increased awareness, because we already do a lot of things. (14)

Furthermore, healthcare professionals indicated having focused more on small things that matter to the residents due to WINC. A healthcare professional added that she appreciated that more focus was given to well-being, instead of just physical matters. A member of the local multidisciplinary working group indicated that contact between the care team and the well-being coach increased, which was reported as positive:

I feel a lot of nice little things were done as a result of WINC. And that you are also aware of doing it together. What would the resident like and how does someone retain their self-worth? A lot of colleagues are very creative in that. (03)

#### Outcome measures

**Figure 2.**
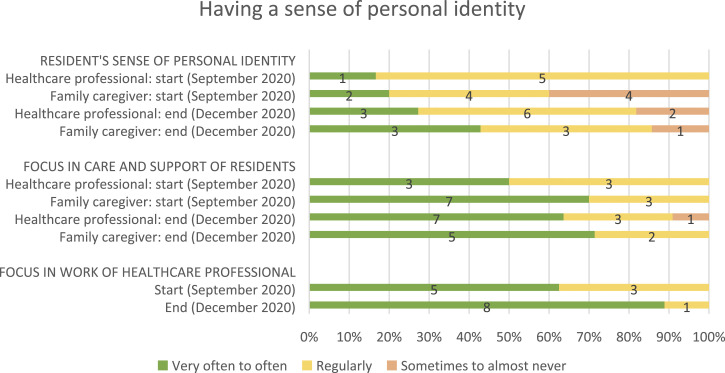
Resident’s sense of personal identity (from family caregivers and healthcare professionals perspectives), its focus in residents’ care and support, and its focus in the work of healthcare professionals. For healthcare professionals, data from 10 and 8 of 18 were missing at the start and end, respectively. For family caregivers, data from 3 and 6 of 13 was missing at the start and end, respectively. And for proxy questionnaires filled in by healthcare professionals about the person living with dementia, data was missing from 8 and 3 of 14 at the start and end, respectively.

**Figure 3.**
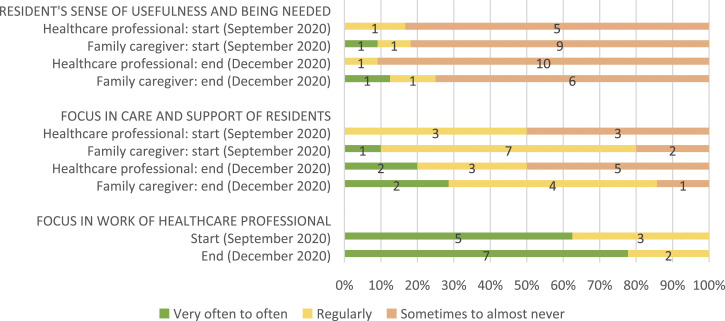
Resident’s sense of usefulness and being needed (from family caregivers and healthcare professionals perspectives), its focus in residents’ care and support, and its focus in the work of healthcare professionals. For healthcare professionals, data from 10 and 8 of 18 were missing at the start and end, respectively. For family caregivers, data from 3 and 6 of 13 was missing at the start and end, respectively. And for proxy questionnaires filled in by healthcare professionals about the person living with dementia, data was missing from 8 and 3 of 14 at the start and end, respectively.

**Figure 4.**
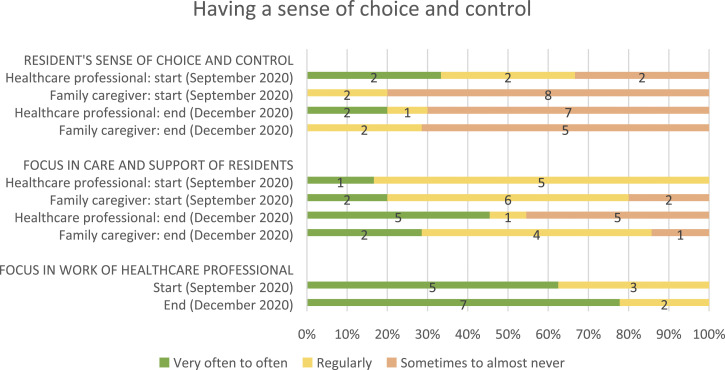
Resident’s sense of choice and control (from family caregivers and healthcare professionals perspectives), its focus in residents’ care and support, and its focus in the work of healthcare professionals. For healthcare professionals, data from 10 and 8 of 18 were missing at the start and end, respectively. For family caregivers, data from 3 and 6 of 13 was missing at the start and end, respectively. And for proxy questionnaires filled in by healthcare professionals about the person living with dementia, data was missing from 8 and 3 of 14 at the start and end, respectively.

**Figure 5.**
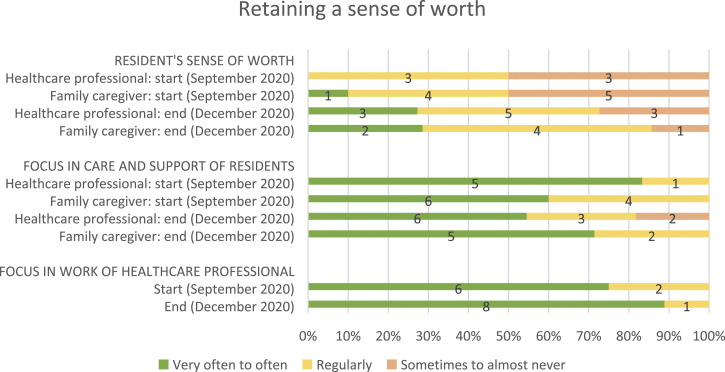
Resident’s sense of worth (from family caregivers and healthcare professionals perspectives), its focus in residents’ care and support, and its focus in the work of healthcare professionals. For healthcare professionals, data from 10 and 8 of 18 were missing at the start and end, respectively. For family caregivers, data from 3 and 6 of 13 was missing at the start and end, respectively. And for proxy questionnaires filled in by healthcare professionals about the person living with dementia, data was missing from 8 and 3 of 14 at the start and end, respectively.

Table 2 shows all of the outcome measures for the starting and follow-up measurements. Healthcare professionals and family caregivers also reported on residents’ feelings of empowerment, how much focus was on empowerment in the care and support of residents, and how much focus there was on empowerment in the daily work of healthcare professionals (see Figure 2, 3, 4 and 5, respectively).

**Table 2.** Changes after implementing WINC intervention on the primary and secondary outcome measures for the person living with dementia (n = 13), their family caregiver (n = 14) and healthcare professionals (n = 18).

	Start	End
	Median (range)	Median (range)
Resident living with dementia^*^		
Quality of life (TOPICS-MDS)		
Proxy perspective of residents by HCP	4.5 (3–7)^d^	6.0 (3–8)^a^
Proxy perspective of residents by family caregiver	6.0 (3–8)^e^	7.0 (5–8)^g^
Perspective of HCP	5.5 (3–6)^d^	5.0 (3–7)^a^
Perspective of family caregiver	5.0 (3–8)^e^	6.0 (3–7)^g^
Health-related quality of life (TOPICS-MDS)		
Proxy perspective of residents by HCP	5.0 (3–7)^d^	5.0 (4–9)^a^
Proxy perspective of residents by family caregiver	7.0 (3–7)^f^	7.0 (4–8)^g^
Perspective of HCP	6.0 (3–8)^d^	6.0 (5–8)^a^
Perspective of family caregiver	6.0 (3–8)^e^	6.0 (3–7)^g^
Behaviour		
Apathy (AES-10)	28.0 (15–36)^d^	32.0 (17–39)^a^
Challenging behaviour (NPI-Q)	15.5 (0–47)^d^	11.0 (0–75)^a^
Mood (NORD)	3.0 (0–4)^d^	2.0 (1–4)^c^
Social engagement (RISE)	2.0 (0–6)^d^	4.0 (0–6)^b^
Family caregiver ^^^		
Quality of life (TOPICS-MDS)	7.5 (6–9)^e^	7.0 (6–9)^g^
Health-related quality of life (TOPICS-MDS)	3.0 (1–4)^e^	3.0 (1–4)^g^
Caregiver quality of life (carer-QoL)	88.3 (59.3–100)^f^	84.7 (73.8–95.4)^g^
Sense of competence (SSCQ)	31.5 (24–35)^e^	34.0 (20–35)^h^
Caregiver burden (TOPICS-MDS)	4.5 (0–7.5)^e^	2.0 (0–6)^g^
Healthcare professional ^+^		
Job satisfaction (LQWQ)	20.0 (17–21)^j^	18.5 (17–22)^j^
Job demands (LQWQ)	14.5 (13–16)^j^	15.0 (12–17)^i^
Team climate (TCI)	76.5 (66–82)^j^	77.0 (68–87)^i^

HCP = healthcare professionals.

* For people living with dementia, higher scores indicate better (health-related) quality of life, more apathy, more challenging behaviour, more depressive symptoms, and more social engagement.

^ For family caregivers, higher scores indicate better (health-related) quality of life, a better care situation, and a higher sense of competence.

^+^ For healthcare professionals, higher scores indicate that nurses perceive their job satisfaction and job demands as more positive, and indicate a more positive team climate.

^a^ 3, ^b^ 4, ^c^ 5, ^d^ 8 of 14 missing, respectively. ^e^ 3, ^f^ 4, ^g^ 6, ^h^ 7 of 13 missing, respectively. ^I^ 9, ^j^ 10 of 18 missing, respectively.

## Discussion

This study described the development and feasibility evaluation of the WINC empowerment program. It showed that, according to healthcare professionals, the newly developed empowerment program was feasible for promoting empowerment in people living with dementia in a nursing home. The program was considered practical and suitable to their way of working. Nevertheless, the enthusiasm that healthcare professionals had about the program, and their feelings of its added value, varied. Regarding the implementation, healthcare professionals experienced difficulties in involving family caregivers in the program, and felt that a lack of time hindered their focus on the themes of empowerment. Yet, some healthcare professionals also mentioned after using the empowerment program, having an increased awareness regarding the four themes of empowerment, and gave greater focus on the small things that mattered to residents. Responses to the questionnaires showed no improvement on the self-reported focus on empowerment for healthcare professionals and the feelings of empowerment of residents from the perspectives of the healthcare professionals and family caregivers. However, this might not be a valid reason for the research team to discontinue the further development and evaluation, as due to the hindrance of the COVID-19 outbreak, results should be interpreted with caution. During the COVID-19 pandemic, healthcare professionals had to work under complex and stressful circumstances (Snyder et al., 2021; White et al., 2021). Considering the high workload due to COVID-19 related priorities, the lower number of healthcare professionals who were able to participate, and COVID-19 restrictions, such as a maximum number of healthcare professionals that could attend a meeting, results could have been negatively affected.

The local multidisciplinary working group was of added value for the implementation of the program, as it helped to adjust the WINC empowerment program to fit the local setting and working routines. However, the role of family caregivers requires extra attention in the future, as involving family caregivers was challenging, and healthcare professionals highlighted the added value of family caregivers taking a more active role in formulating specific goals for the resident. Previous studies have reported on the challenges of family involvement (Puurveen et al., 2018; Reid & Chappell, 2017; Tasseron-Dries et al., 2021). Involvement may differ between family caregivers, as it depends on the degree to which family caregivers consider their own involvement to be important (Reid & Chappell, 2017). Healthcare professionals may have a crucial leadership role in demonstrating mutual recognition and respect through the creation of welcoming environments that enable the family to participate, the provision of adequate information, and enacting collaborative relationships (Puurveen et al., 2018). Meaningful family involvement may be established by clear communication about mutual expectations, with an emphasis on the benefits for both the resident and family caregiver (Tasseron-Dries et al., 2021). Future research should, together with all stakeholders, investigate how family caregivers can be included and feel motivated to be involved in the WINC project.

Furthermore, it is interesting to note that the modules that are not incorporated in normal working routines (such as the Empowerment Café and observation of a colleague) were perceived more positively than modules that fall within normal working routines (such as the exercise to focus on the themes of empowerment). This is possibly not surprising, as changing daily routines can be more disruptive or difficult. Another explanation for this could be that the modules outside of the daily routines were performed together with colleagues, in contrast to individual exercises. This could have increased enthusiasm and motivation (Willemse et al., 2012), which would advocate for emphasising collaboration and shared experiences between healthcare professionals during the WINC empowerment program. During this feasibility evaluation, the sharing of experiences was hindered due to COVID-19 restrictions. It is apparent that pressures on communication, teamwork, staffing and time are barriers to implementation of the WINC empowerment program into daily routines (Lawrence et al., 2012). Policy makers and managers of long-term care organizations could benefit from embracing the promising effects of empowerment through the facilitation of necessary prerequisites. For example by encouraging healthcare professionals to take time to connect with people living with dementia, their family caregivers, and for dialogue and reflection with colleagues. Moreover, in the development phase of the program, based on the Intervention Mapping methodology, we identified behaviour change techniques that are suitable for nursing home staff in their current daily routines. Through this, we aimed to facilitate healthcare professionals in promoting empowerment for residents in their daily work.

### Strengths and limitations

To our knowledge, this is the first study to develop and evaluate an intervention specifically aimed at promoting empowerment in people living with dementia in nursing homes. A strength of the study is the evidence-based methods used in the development and feasibility evaluation of the intervention. The combination of quantitative and qualitative data collection provided valuable insights into the feasibility. A limitation of this study is the potential selection bias towards motivated care teams, as participation was done by invitation. Yet, motivation was seriously hindered by the consequences of the COVID-19 pandemic. The study had to stop, and restart 6 months later, which took increased effort to regain the motivation and focus of healthcare professionals during the restart. Also, the results on the limited efficacy of the program could be biased, as changes in the COVID-19 situation may have influenced outcome measures. Lastly, not all questionnaires were completed by all participants, and not all pre-planned ocus groups discussions could take place due to COVID-19 restrictions, which caused a more limited sample size. Since firm conclusions cannot be drawn due to these limitations, more research is needed to substantiate our results.

### Further research

Based on the experiences of healthcare professionals, we will optimise the empowerment program in a refined intervention by addressing the issues from this evaluation, such as promoting collaboration between healthcare professionals and the involvement of family caregivers. It is useful to include multiple stakeholders in this refinement process. Also, the program may benefit from addressing ways promoting empowerment for multiple residents at the same time, as healthcare professionals perceived this as advisable yet difficult. Our study showed that healthcare professionals experienced a lack of time as a barrier, and this suggests that having more staff available might encourage healthcare professionals to support residents to complete tasks themselves instead of taking over these tasks. This might contribute to promoting empowerment. However, it seems important that the WINC empowerment program is feasible within available resources. Therefore, it is important to further investigate the feasibility. More information is needed about the refined intervention, and its feasibility in a non-pandemic situation. Thereafter, following the MRC framework, future research may be undertaken to investigate the effects of the program by means of a randomised controlled trial.

## Conclusion

This study shows that the WINC empowerment program is a feasible intervention for healthcare professionals to promote empowerment in residents living with dementia. An important step is to take into account implementation prerequisites that follow from the findings of this study, and accordingly, further investigate the effects of the WINC empowerment program on feelings of empowerment within residents, and the changes in awareness, attitudes and behaviour of healthcare workers towards an empowerment-promoting approach.

## Supplemental Material

Supplemental Material - Promoting empowerment for people living with dementia in nursing homes: Development and feasibility evaluation of an empowerment program Click here for additional data file. Supplemental Material for Promoting empowerment for people living with dementia in nursing homes: Development and feasibility evaluation of an empowerment program by Charlotte van Corven, Annemiek Bielderman, Mandy Wijnen, Ruslan Leontjevas, Maud JL Graff and Debby L Gerritsen in Dementia
