# Supplementary material for: Promoting empowerment for people living with dementia in nursing homes: Development and feasibility evaluation of an empowerment program
Source: Dementia (London). 2022 Sep 5;21(8):2517–35. doi: 10.1177/14713012221124985 (PMC9583290; doi:10.1177/14713012221124985)
Supplement: Supplemental Material - Promoting empowerment for people living with dementia in nursing homes: Development and feasibility evaluation of an empowerment program [file sj-pdf-1-dem-10.1177_14713012221124985.pdf]

Additional File 1. Feasibility results at initial start, restart and end

Table 1. Demand of WINC

Healthcare professionals	Start	Restart
I would like to work with WINC	16 of 16[a]	7 of 8[b]
I think my colleagues would like to work with WINC	12 of 16[a]	7 of 8[b]

[a] 2, [b] 10 of 18 missing

Table 2. Acceptability of WINC

Healthcare professionals – expectations	Start	Restart
My first impression of WINC is good	14 of 16[a]	8 of 8[b]
I am motivated to work with WINC	15 of 16[a]	8 of 8[b]
I expect to enjoy working with WINC together with my colleagues	16 of 16[a]	7 of 8[b]
I expect that WINC is of value to my job as a healthcare professional	14 of 16[a]	7 of 8[b]
I expect that WINC is of value to the care and support of residents	15 of 16[a]	7 of 8[b]

Family caregivers – expectations	Start	Restart
My first impression of WINC is good	7 of 8[c]	5 of 10[e]
I think it is a good idea that the nursing homes will work with WINC	7 of 7[d]	8 of 10[e]
I think WINC will be of value to the care and support of residents	6 of 8[c]	7 of 10[e]

Healthcare professionals – experiences	End
I enjoyed working with WINC	4 of 8[b]
WINC was of value to my job as a healthcare professional	4 of 8[b]
WINC was of value to the care and support of residents	3 o f8[b]
I would like to keep using WINC in the future	3 of 8[b]
I would advise other teams in my organisation to work with WINC	4 of 8[b]

Family caregivers - experiences	End
I was well-informed about WINC	3 of 7[f]
WINC was of value to the care and support of residents	2 of 7[f]
I was involved enough in WINC	4 of 7[f]

[a] 2, [b] 10 of 18 missing
[c] 5, [d] 6, [e] 3, [f] 6 of 13 missing

Table 3. Implementation

Healthcare professionals	End
I’ve shared my experiences with WINC with colleagues	7 of 9[a]

[a] 9 of 18 missing

Table 4. Practicality

Healthcare professionals – expectations	Start	Restart
I have enough time to work with WINC	8 of 16[a]	3 of 8[b]
It is clear what is expected of me during WINC		8 of 8[b]

Healthcare professionals – experiences	End
I had enough time to work with WINC	3 of 9[c]
It was clear what was expected of me during WINC	7 of 9[c]

[a] 2, [b] 10, [c] 9 of 18 missing

Additional File 2. Outcome measures

Person living with dementia

Themes of empowerment

The four themes of empowerment were assessed by the family caregiver and healthcare professional, whereby they questioned the feelings of empowerment for each theme and how much attention was given to empowerment in the care and support of the resident. Questions were answered on a five-point Likert scale from ‘almost never’ to ‘always’.

(Health-related) Quality of life

Quality of life and health-related quality of life was assessed through proxy and proxy-proxy with the TOPICS-MDS by the family caregiver and healthcare professional (Lutomski et al., 2013). Participants rated the residents (health-related) quality of life from 0 to 10. Higher scores indicate a higher (health-related) quality of life.

Neuropsychiatric symptoms

Neuropsychiatric symptoms were assessed using the Neuropsychiatric Inventory nursing home version (NPI-NH) (Cummings et al., 1994). In the NPI-NH, twelve neuropsychiatric symptom domains are assessed by healthcare professionals: delusions, hallucinations, agitation or aggression, dysphoria or depression, anxiety, euphoria or elation, apathy or indifference, disinhibition, irritability or lability, aberrant motor behaviours, night-time behavioural disturbances, and appetite or eating disturbances. A screening question determines if the behaviour is present (yes or no). If the symptom is present, both frequency (F) and severity (S) are scored on a four-point and three-point Likert scale, respectively. By multiplying the F and S score (F × S), a separate score can be calculated, resulting in values ranging from 0 to 12 points per symptom. The sum of the F × S score for each symptom provides a total score that ranges from 0 to 144. A higher score indicates a higher frequency and/or severity of neuropsychiatric symptoms.

Initiative

Apathy was examined using the abbreviated Apathy Evaluation Scale (AES-10) (Lueken et al., 2007). Scores could range from 10 to 40, and higher scores indicate more apathy. This 10-item observational scale was completed by the healthcare professional. The healthcare professional evaluates to what degree a specific apathetic behaviour is characteristic of the resident. The four response categories range from ‘not at all characteristic’ to ‘very characteristic’. Total scores can range from 10 to 40. Higher scores indicate more apathetic behaviour. Scores were invalid if more than one answer was missing (Leontjevas, Evers-Stephan, et al., 2012; Lueken et al., 2007).

Mood

Mood was assessed by the healthcare professional using the Nijmegen Observer-Rated Depression scale (NORD). The NORD consists of five questions regarding depressive behaviours, rated as either absent or present. The sum of present symptoms provides a total score that ranges from 0 to 5, with higher scores indicating more depressive symptoms. Total scores were invalid if one or more answers were missing (Leontjevas, Gerritsen, et al., 2012)

Social engagement

Social engagement was assessed by the healthcare professional using the Revised Index for Social Engagement for Long-Term Care (RISE). The RISE consists of six questions regarding social engagement, rated as either absent or present. The sum of present symptoms provides a total score that ranges from 0 to 6, with higher scores indicating more social engagement. Total scores were invalid if one or more answers were missing (Gerritsen et al., 2008).

Family caregivers

The perceived quality of life and health-related quality of life of family caregivers was measured using the TOPICS-MDS question on a scale from 1 to 5. Subjective caregiving burden was assessed by the carer-QoL. The carer-QoL comprises of seven items with three response categories that range from 'none' to 'much'. The visual analogue scale ranged from 0 to 10, labelled from 'not heavy at all' to 'way too heavy', to rate a caregiver's level of burden in providing care and support. Total scores could range from 0-100, and scores were invalid if more than one answer was missing. Higher scores indicated a better care situation (Brouwer et al., 2006; Melis et al., 2019).

To examine the caregivers' sense of competence, the Short Sense of Competence Questionnaire (SSCQ) was used (Vernooij-Dassen et al., 1999). This scale assesses the family caregivers' feelings of capability in caring for a person with dementia. The SSCQ totals seven items with five response categories that range from 'agree very strongly' to 'disagree very strongly'. The SSCQ has satisfactory validity and reliability (Vernooij-Dassen et al., 1999). Total scores could range from 0 to 7, and scores were invalid if more than one answer was missing. A higher score indicated a greater sense of competence.

Healthcare professionals

Job satisfaction and job demands

Job satisfaction and job demands were assessed with two subscales of the Leiden Quality of Work Questionnaire (van der Doef & Maes, 1999). This questionnaire is a validated and reliable instrument assessing thirteen job characteristics of nursing staff (van der Doef & Maes, 1999). Job satisfaction consists of seven items (range 7–28) and job demands has six items (range 6–24). Questions are answered on a four-point Likert scale ranging from 'totally disagree' (1) to 'totally agree' (4). A higher score indicates that nurses perceive their job satisfaction and job demands as more positive. Scores were invalid if more than one answer was missing.

Team climate

Team climate was assessed with two subscales of the Team Climate Inventory: participative safety and support for innovation (Anderson & West, 1998). This Dutch version of the questionnaire is a validated and reliable instrument (Ouwens et al., 2009), and has been used for healthcare professionals in nursing homes previously (Heponiemi et al., 2012). Questions are answered on a five-point Likert from 'totally disagree' (1) to 'totally agree' (5). A higher score indicates a more positive team climate. Total scores could range from 20 to 100, and scores were invalid if more than one answer was missing.

References

Anderson, N. R., & West, M. A. (1998). Measuring climate for work group innovation: development and validation of the team climate inventory. *Journal of Organizational Behavior: The International Journal of Industrial, Occupational and Organizational Psychology and Behavior*, *19*(3), 235-258. https://doi.org/https://doi.org/10.1002/(SICI)1099-1379(199805)19:3<235::AID-JOB837>3.0.CO;2-C

Brouwer, W. B., van Exel, N. J., van Gorp, B., & Redekop, W. K. (2006). The CarerQol instrument: a new instrument to measure care-related quality of life of informal caregivers for use in economic evaluations. *Qual Life Res*, *15*(6), 1005-1021. https://doi.org/10.1007/s11136-005-5994-6

Cummings, J. L., Mega, M., Gray, K., Rosenberg-Thompson, S., Carusi, D. A., & Gornbein, J. (1994). The Neuropsychiatric Inventory: comprehensive assessment of psychopathology in dementia. *Neurology*, *44*(12), 2308-2314. https://doi.org/10.1212/wnl.44.12.2308

Gerritsen, D. L., Steverink, N., Frijters, D. H., Hirdes, J. P., Ooms, M. E., & Ribbe, M. W. (2008). A revised Index for Social Engagement for long-term care. *J Gerontol Nurs*, *34*(4), 40-48. https://doi.org/10.3928/00989134-20080401-04

Heponiemi, T., Elovainio, M., Kouvonen, A., Noro, A., Finne-Soveri, H., & Sinervo, T. (2012). Ownership type and team climate in elderly care facilities: the moderating effect of stress factors. *J Adv Nurs*, *68*(3), 647-657. https://doi.org/10.1111/j.1365-2648.2011.05777.x

Leontjevas, R., Evers-Stephan, A., Smalbrugge, M., Pot, A. M., Thewissen, V., Gerritsen, D. L., & Koopmans, R. T. (2012). A comparative validation of the abbreviated Apathy Evaluation Scale (AES-10) with the Neuropsychiatric Inventory apathy subscale against diagnostic criteria of apathy. *J Am Med Dir Assoc*, *13*(3), 308.e301-306. https://doi.org/10.1016/j.jamda.2011.06.003

Leontjevas, R., Gerritsen, D. L., Vernooij-Dassen, M. J., Teerenstra, S., Smalbrugge, M., & Koopmans, R. T. (2012). Nijmegen observer-rated depression scale for detection of depression in nursing home residents. *Int J Geriatr Psychiatry*, *27*(10), 1036-1044. https://doi.org/10.1002/gps.2819

Lueken, U., Seidl, U., Völker, L., Schweiger, E., Kruse, A., & Schröder, J. (2007). Development of a short version of the Apathy Evaluation Scale specifically adapted for demented nursing home residents. *The American journal of geriatric psychiatry*, *15*(5), 376-385. https://doi.org/https://doi.org/10.1097/JGP.0b013e3180437db3

Lutomski, J. E., Baars, M. A., Schalk, B. W., Boter, H., Buurman, B. M., den Elzen, W. P., Jansen, A. P., Kempen, G. I., Steunenberg, B., & Steyerberg, E. W. (2013). The development of the Older Persons and Informal Caregivers Survey Minimum DataSet (TOPICS-MDS): a large-scale data sharing initiative. *PLoS One*, *8*(12), e81673. https://www.ncbi.nlm.nih.gov/pmc/articles/PMC3852259/pdf/pone.0081673.pdf

Melis, R. J. F., van Hout, H. P. J., & Metzelthin, S. F. (2019). The Older Persons and Informal Caregivers Survey Minimum Dataset (TOPICS-MDS). In D. Gu & M. E. Dupre (Eds.), *Encyclopedia of Gerontology and Population Aging* (pp. 1-9). Springer International Publishing. https://doi.org/10.1007/978-3-319-69892-2_981-1

Ouwens, M., Hulscher, M., & Wollersheim, H. (2009). Meten van teamklimaat. *Kwaliteit in zorg*, *4*, 14-18.

van der Doef, M., & Maes, S. (1999). The Leiden Quality of Work Questionnaire: its construction, factor structure, and psychometric qualities. *Psychological reports*, *85*(3), 954-962. https://doi.org/https://doi.org/10.2466/pr0.1999.85.3.954

Vernooij-Dassen, M. J., Felling, A. J., Brummelkamp, E., Dauzenberg, M. G., van den Bos, G. A., & Grol, R. (1999). Assessment of caregiver's competence in dealing with the burden of caregiving for a dementia patient: a Short Sense of Competence Questionnaire (SSCQ) suitable for clinical practice. *J Am Geriatr Soc*, *47*(2), 256-257. https://doi.org/10.1111/j.1532-5415.1999.tb04588.x

Additional File 3. Intervention mapping step 2

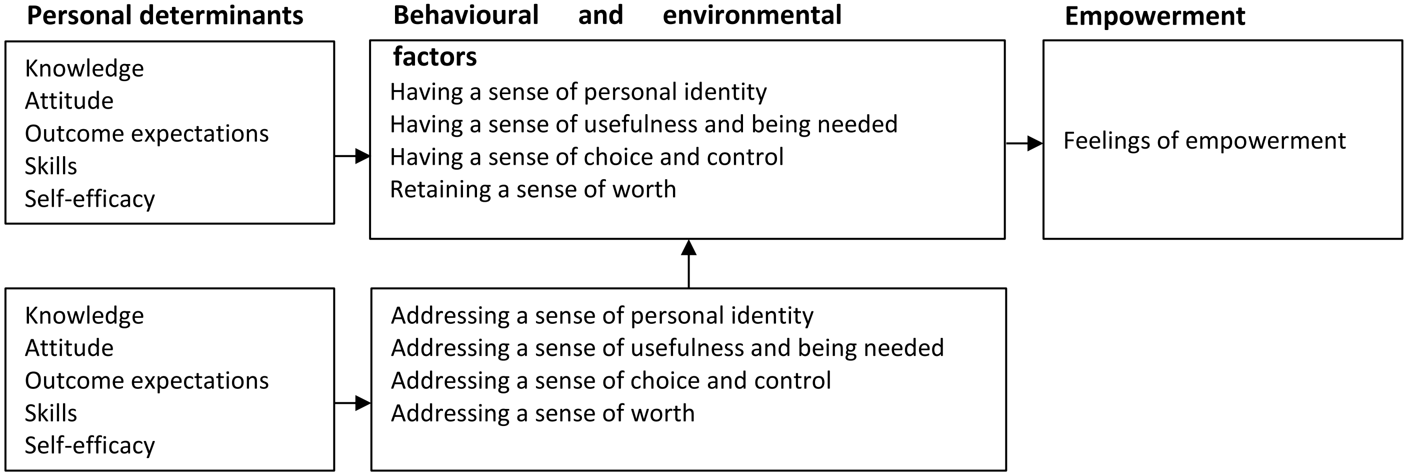

Figure 1. Logic model of change.

Table 1. Individual matrix of change objectives

	Knowledge	Attitude	Outcome expectations	Skills	Self-efficacy
Having a sense of personal identity	Describe components to be the person you are	Recognise the advantages of being the person you are	Expect that being the person you are will increase well-being	Demonstrate the ability to be the person you are	Express confidence in the ability to be the person you are
Having a sense of usefulness and being needed	Identify methods to have a sense of usefulness and being needed	Recognise the advantages of having a sense of usefulness and being needed	Expect that having a sense of usefulness and being needed will increase well-being	Demonstrate the ability to have a sense of usefulness and being needed	Express confidence in the ability to have a sense of usefulness and being needed
Having a sense of choice and control	Identify methods of making choices and having control	Recognise the advantages of making choices and having control	Expect that making choices and having control will increase well-being	Demonstrate ability to make choices and have control	Express confidence in the ability to make choices and have control
Retaining my sense of worth	List the steps to successfully retain my sense of worth	Recognise the advantages of retaining my sense of worth	Expect that retaining my sense of worth will increase well-being	Demonstrate the ability to retain my sense of worth	Express confidence in the ability to retain my sense of worth

Table 2. Environmental matrix of change objectives

	Knowledge	Attitude	Outcome expectations	Skills	Self-efficacy
Having a sense of personal identity	Know methods of promoting a sense of personal identity	Recognise the advantages of promoting a sense of personal identity	Expect that promoting a sense of personal identity will increase well-being	Demonstrate ability to promote a sense of personal identity	Express confidence in the ability to promote a sense of personal identity
Having a sense of usefulness and being needed	Know methods of promoting a sense of usefulness or being needed	Recognise the advantages of promoting a sense of usefulness or being needed	Expect that promoting a sense of usefulness or being needed will increase well-being	Demonstrate ability to promote a sense of usefulness or being needed	Express confidence in the ability to promote a sense of usefulness or being needed
Having a sense of choice and control	Know methods of promoting a sense of choice and control	Recognise the advantages of promoting a sense of choice and control	Expect that promoting a sense of choice and control will increase well-being	Demonstrate ability to promote a sense of choice and control	Express confidence in the ability to promote a sense of choice and control
Retaining a sense of worth	Know methods of promoting a sense of self-worth	Recognise the advantages of promoting a sense of self-worth	Expect that promoting a sense of self-worth will increase well-being	Demonstrate ability to promote a sense of self-worth	Express confidence in the ability to promote a sense of self-worth

Additional File 4. Intervention mapping step 3

Behavioural change techniques:

- **Promoting focus on past successes** = involves instructing the person to think about or list previous successes in performing the behaviour (or parts of it) (Michie et al., 2011 – 18)
- **Facilitate social comparison** = involves explicitly drawing attention to others' performance to elicit comparisons (Michie et al., 2011 – 28)
- **Motivational interviewing** = this is a clinical method including a specific set of techniques involving prompting the person to engage in change talk in order to minimise resistance and resolve ambivalence to change (includes motivational counselling) (Michie et al., 2011 – 37)
- **Action planning** = involves detailed planning of what the person will do including, as a minimum, when, in which situation and/or where to act (Michie et al., 2011 – 7)
- **Plan social support / social change** = involves prompting the person to plan how to elicit social support from other people who help them achieve their target behaviour or outcome (Michie et al., 2011 – 29)
- **Barrier identification / problem solving** = this presumes having formed an initial plan to change behaviour. The person is prompted to think about potential barriers and identify ways of overcoming them (Michie et al., 2011 – 8)
- **Repeated exposure** = making a stimulus repeatedly accessible to the individual's sensory receptors.
- **Provide instruction on how to perform the behaviour** = involves telling the person how to perform the behaviour or preparatory behaviours, either verbally or in written form (Michie et al., 2011 – 21)
- **Provide information on consequences of the behaviour** = information about the relationship between the behaviour and its possible or likely consequences (Michie et al., 2011 – 1,2)

Table 3 illustrates how and which behaviour change techniques were chosen to address the determinants.

Table 3. Behaviour change techniques of determinants of behaviour

Determinants of behaviour	Behaviour change techniques	Application
Knowledge: know methods of empowering a person with dementia	Provide instruction on how to perform the behaviour<br>Provide information on consequences of the behaviour	M1: Empowerment Café
Attitude: recognise the advantages of empowering a person with dementia	Provide information on consequences of the behaviour<br>Active learning<br>Direct experience<br>Facilitate social comparison<br>Goal setting (behaviour and outcome)	M1: Empowerment Café<br>M2: Observe a colleague<br>M2: Focus on themes<br>M3: Empowerment for residents
Outcome expectations: expects that empowering a person with dementia will increase well-being	Provide information on consequences of the behaviour<br>Motivational interviewing<br>Active learning<br>Direct experience<br>Goal setting (behaviour and outcome)<br>Prompt review of behavioural and outcome goals<br>Prompting self-monitoring of behaviour / outcome	M1: Empowerment Café<br>M2: Observe a colleague<br>M2: Focus on themes<br>M3: Empowerment for residents
Skills: demonstrate the ability to empower a person with	Provide instruction on how to perform the behaviour	M2: Observe a colleague<br>M2: Focus on themes

dementia	Active learning<br>Direct experience<br>Prompt practice<br>Prompting generalisation of target behaviour	M3: Empowerment for residents
Self-efficacy: express confidence in the ability to empower a person with dementia	Motivational interviewing<br>Prompting focus on past successes<br>Provide feedback on performance<br>Direct experience<br>Self-affirmation<br>Prompt review of behavioural and outcome goals<br>Prompting self-monitoring of behaviour / outcome<br>Prompting generalisation of target behaviour	M1: Empowerment Café<br>M2: Observe a colleague<br>M2: Focus on themes

Other behavioural change methods used throughout the whole program are: (1) repeated exposure, (2) barrier identification / problem, (3) action planning, (4) plan social support / social change, and (5) relapse prevention / coping planning.

Additional File 5. Evaluation phase results per module

Acceptability

Expectations before the start of WINC

In questionnaires at the initial start in March 2020 for the first module specifically, 15/16 healthcare professionals expected it to be useful to discuss with colleagues the themes of empowerment, and expected the Empowerment Café to be of value to their job as a healthcare professional.

For the second module, 8/8 healthcare professionals indicated that they expected to enjoy observing a colleague, and expected that this would be valuable for their job as a healthcare professional (same in March 2020). Furthermore, 7/8 healthcare professionals reported that it seemed useful to them to reflect on this observation using their personal booklet (8/8 in March 2020), and 8/8 indicated this appraisal for sharing experiences with colleagues (same in March 2020). Furthermore, 7/8 healthcare professionals indicated that they expected to enjoy the exercise of focusing on the four themes of empowerment, and expected that this would be of value for their job as a healthcare professional (8/8 in March 2020). Furthermore, 7/8 healthcare professionals reported that it seemed useful to them to reflect on this exercise using their personal booklet, and by sharing experiences with colleagues (8/8 in March 2020).

Experiences using WINC

In follow-up questionnaires for the first module, 6/7 healthcare professionals indicated that they found it beneficial to discuss the themes of empowerment with colleagues, 8/8 noted that there was a good atmosphere, and 5/7 reported that the Empowerment Café was of value for their work as a healthcare professional (7/8 in March 2020). 6/7 healthcare professionals reported that the Empowerment Café made them motivated to work with WINC (8/8 in March 2020).

For the second module, 6/6 healthcare professionals reported enjoying observing a colleague, and 5/6 indicated that this was valuable to their work as a healthcare professional. 3/5 healthcare professionals indicated that reflecting on this by means of questions in their personal booklet was useful. Furthermore, 3/7 healthcare professionals stated that they enjoyed the exercise in which they focused on the themes of empowerment in their daily work, and found this beneficial for their work as a healthcare professional. 3/6 healthcare professionals stated that reflecting on this by means of questions in a personal booklet was useful. During the interviews, healthcare professionals highlighted the usefulness of observing a colleague. Nevertheless, some mentioned that they felt nervous or insecure when a colleague observed them.

With regards to the third module, 7/7 healthcare professionals reported enjoying reflecting on the themes of empowerment for each resident in a small multidisciplinary group, and 3/6 indicated this was of value for the care and support of residents. Furthermore, 4/6 healthcare professionals reported enjoying working on the specific goals that arose from the meetings.

Implementation

From the field notes, for the first module specifically, 25 people participated in one of the two Empowerment Cafés, which means that all care professionals in the participating teams were present during an Empowerment Café (except one due to illness).

In the questionnaires, for the second module, 6/9 healthcare professionals indicated having observed a colleague, nurse, nursing assistant or well-being coach. From the interviews and field notes, it appeared to differ between the healthcare professionals if, during the observation, the focus was on the four themes of empowerment, or more general, such as the work of other

disciplines or the organisation. The three healthcare professionals who reported that they did not observe a colleague stated COVID-19 as the reason. Only one of them specified this in the questionnaire, as she indicated, due to the COVID-19 restrictions, she could not observe a colleague from another location, which she would have liked to have done so. Observation ranged from 1-4 hours. 5/6 healthcare professionals reported filling in the reflection questions in their personal booklet afterwards. Furthermore, 7/9 healthcare professionals indicated that they performed the exercise in which they focused on each theme of empowerment during their daily work. The two healthcare professionals who indicated that they did not noted COVID-19 as the reason, but did not provide specification on this. 6/6 healthcare professionals filled in the reflection questions in their personal booklet afterwards.

Regarding the third module, 7/9 healthcare professionals reported attending a multidisciplinary meeting in which reflection on each theme of empowerment per resident was considered. From the field notes and interviews, it emerged that some healthcare professionals did not attend due to the COVID-19 restrictions as the group would have been too big with their presence. In the questionnaires, 4/6 healthcare professionals indicated having actively worked on the specific goals that arose from the meetings, and 5/6 shared their experiences of working on these goals with colleagues. During the interviews, possible strategies mentioned by healthcare professionals for implementing the specific goals included linking activities to certain shifts, or to specifically plan them for yourself. Healthcare professionals indicated reporting these goals in the resident's care plans. These goals were reported for all participating residents. From the care plans, it emerged that most goals pertained to pleasurable or meaningful activities, or actions to promote comfort. From the field notes and interviews, it was apparent that other healthcare professionals read the specific goals. Nevertheless, disciplines who work with many residents mentioned in interviews that they do not read all of the care plans. They only worked with the goals when others specifically asked them. The well-being coach reported to be actively involved in putting specific goals for residents into practice.

For the fourth module, only one Empowerment Café was held, with seven participants, as the other Empowerment Café's were cancelled due to new COVID-19 measures.

Practicality

Expectations before the start of WINC

In questionnaires prior to the start, 13/16 healthcare professionals felt they had enough time to attend the Empowerment Café, 7/8 to observe a colleague (same in March 2020), while only 3/8 felt they had enough time to work with the exercises focusing on the themes of empowerment in their daily work (4/8 in March 2020).

Experiences using WINC

In the follow-up questionnaires for the first module, 6/7 healthcare professionals indicated that the provided information was not difficult, and 8/8 noted that the duration and location of the Empowerment Café was good.

For the second module, 2/7 felt they had enough time to perform the exercises to focus on the themes of empowerment, 4/6 said they felt they had enough time to observe a colleague. During the focus group interview, a healthcare professional noted that observing colleagues can also cause distress for residents as they see multiple new faces.

Regarding the third module, 3/6 healthcare professionals noted having enough time to attend the multidisciplinary meetings, and 3/7 reported having enough time to work with the goals formulated

in these meetings. In the interviews, one point for improvement was assigning a moderator (for example a psychologist/team leader) for the meetings, as this may help to structure the meeting.

Limited efficacy

With regards to module 3, when observing colleagues specifically, it appeared from the interviews and field notes to differ for healthcare professionals if, during the observation of a colleague, the focus was on the four themes of empowerment, or more general, such as the work of other disciplines or the organisation as a whole. Moreover, healthcare professionals mentioned that observing a colleague increased the sense of togetherness. They reported that the multidisciplinary meeting helped to get to know residents better, and that reflecting on each resident together with colleagues was inspiring and led to new ideas about care and support. Nevertheless, some healthcare professionals also mentioned they experienced no benefits of the meetings, as they stated that nothing new was discussed.
